# Fanconi syndrome and severe polyuria: an uncommon clinicobiological presentation of a Gitelman syndrome

**DOI:** 10.1186/1471-2431-14-201

**Published:** 2014-08-11

**Authors:** Karim Bouchireb, Olivia Boyer, Lamisse Mansour-Hendili, Arnaud Garnier, Laurence Heidet, Patrick Niaudet, Remi Salomon, Rosa Vargas Poussou

**Affiliations:** 1 Assistance Publique-Hôpitaux de Paris, Service de Néphrologie Pédiatrique, Centre de Référence des Maladies Rénales Héréditaires (MARHEA), Hôpital Necker-Enfants Malades, 149 rue de Sèvres, Paris 75015, France; 2 Service de Néphrologie Pédiatrique et médecine interne, Hôpital d’Enfants, Toulouse, France; 3 Assistance Publique-Hôpitaux de Paris, Service de Génétique, Hôpital Européen Georges Pompidou, Paris, France; 4 Université Paris Descartes, Faculté de Médecine, Paris, France; 5 INSERM, UMR970, centre de recherché cardio-vasculaire, Paris, France

**Keywords:** Polyuria, Polydipsia, Hypokalemia, Fanconi syndrome, Gitelman syndrome

## Abstract

**Background:**

Gitelman syndrome is an autosomal recessive tubulopathy characterized by hypokalemia, hypomagnesemia, metabolic alkalosis and hypocalciuria. The majority of patients do not present with symptoms until late childhood or adulthood, and the symptoms are generally mild. We report here the first case of Gitelman syndrome presenting with the biological features of Fanconi syndrome and an early polyuria since the neonatal period. We discuss in this article the atypical electrolytes losses found in our patient, as well as the possible mechanisms of severe polyuria.

**Case presentation:**

A 6-year-old Caucasian girl was admitted via the Emergency department for vomiting, and initial laboratory investigations found hyponatremia, hypokalemia, metabolic acidosis with normal anion gap, hypophosphatemia, and hypouricemia. Urinalysis revealed Na, K, Ph and uric acid losses. Thus, the initial biological profile was in favor of a proximal tubular defect. However, etiological investigations were inconclusive and the patient was discharged with potassium chloride and phosphorus supplementation. Three weeks later, further laboratory analysis indicated persistent hypokalemia, a metabolic alkalosis, hypomagnesemia, and hypocalciuria. We therefore sequenced the *SLC12A3* gene and found a compound heterozygosity for 2 known missense mutations.

**Conclusions:**

Gitelman syndrome can have varying and sometimes atypical presentations, and should be suspected in case of hypokalemic tubular disorders that do not belong to any obvious syndromic entity. In this case, the proximal tubular dysfunction could be secondary to the severe hypokalemia. This report emphasizes the need for clinicians to repeat laboratory tests in undiagnosed tubular disorders, especially not during decompensation episodes.

## Background

Gitelman syndrome (GOMIM#263800) is an autosomal recessive tubular disorder first described by Gitelman, Graham and Welt in 1966 [1]. It is mainly associated with inactivating mutations in the *SLC12A3* gene located on the long arm of chromosome 16 [2]. This gene encodes the thiazide sensitive sodium chloride co-transporter channel (NCCT) in the distal convoluted tubule. Gitelman syndrome is characterized by hypokalemia, hypomagnesemia, metabolic alkalosis and hypocalciuria. The majority of patients with Gitelman syndrome do not present with symptoms until late childhood or adulthood, and symptoms such as fatigue, vomiting, dizziness, transient episodes of tetany, muscle weakness, cramps are generally mild [3]. Compared with Bartter syndrome in which polyuria is a predominant feature, polyuria and nocturia are less pronounced in Gitelman syndrome [3]. We report herein the observation of unusual presenting features of a genetically confirmed Gitelman syndrome in a 6 year old girl.

## Case presentation

A six year old Caucasian girl was admitted via the pediatric Emergency department with a history of fatigue and vomiting in the previous 24 hours. She was born after an uncomplicated pregnancy obtained by medically assisted procreation. The mother’s medical history was unremarkable. According to the mother, the girl had had polyuria and polydipsia since the neonatal period, with a current daily water intake of 3 liters. On admission, blood pressure was 90/60 mmHg, height was 114 cm (60^th^ percentile), weight was 18 kg (25^th^ percentile). The child had blond hair and blue eyes. She had clinical signs of mild dehydration. Initial laboratory investigations revealed hyponatremia (128 mmol/L), severe hypokalemia (1.6 mmol/L) with electrocardiographic changes, metabolic acidosis (plasma bicarbonates 14 mmol/L) with normal plasma anion gap (14 mmol/L), hypophosphatemia (1 mmol/L), hypouricemia (90 umol/L), normal calcemia (2.26 mmol/L) and a serum creatinine of 28 μmol/l. Urinalysis confirmed the Na+, K + and uric acid losses with inappropriately high Na + (25 mmol/l, fractional excretion of sodium of 3%), K + (85 mmol/l, TTKG 30), tubular phosphate reabsorption rate (18%), and a fractional excretion of uric acid of 30%. Tests for proteinuria and glucosuria were negative. Urinary calcium was not measured initially. Renal ultrasonography showed mild bilateral ureterohydronephrosis. She was rehydrated intravenously with potassium-containing fluid. During hospitalization polyuria was confirmed (2-3 L/day, 4.7-7 L/1.73 m^2^/day). The polyuria, blond hair with blue eyes and some features of Fanconi syndrome were suggestive of cystinosis. However, the leucocyte cystine level was normal. The girl was discharged six days after admission with oral potassium chloride and phosphorus supplementation.

Laboratory studies three weeks after discharge revealed persistent hypokalemia (3 mmol/L), a tendency towards metabolic alkalosis (HCO3^-^ 29 mmol/L), and mild hypomagnesemia (0.65 mmol/L) while serum sodium and serum phosphate were normal. Plasma renin activity was slightly elevated (116 pg/ml). Hypocalciuria was also detected (calciuria 0.34 mmol/l and urinary creatinine 5.24 mmol/l, ratio 0.06 mmol/mmol). Clinically, the child was much less polyuric with urine output of 900 ml per 24 hours.

**Figure 1:**
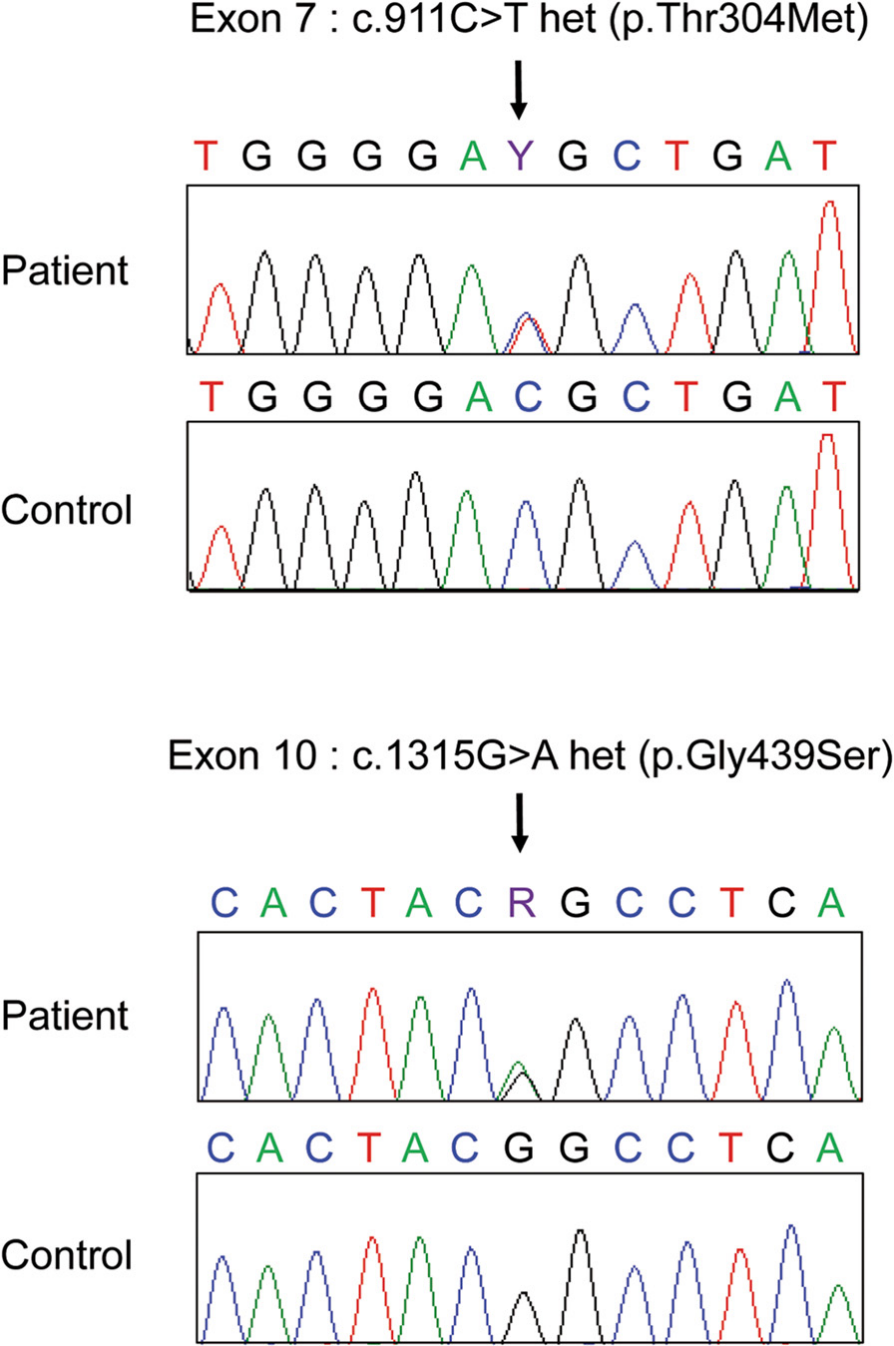
**Sequence analysis of the *SLC12A3 *** **gene revealing a compound heterozygosity for the two previsously published mutations **[4]**,**[5]**.**

The biochemical triad of metabolic alkalosis, hypomagnesemia and hypocalciuria was suggestive of Gitelman syndrome and these findings were confirmed on two other blood and urine analyses. We therefore sequenced the *SLC12A3* gene encoding the NCCT co-transporter and found she was compound heterozygous for 2 known missense mutations (p.Thr304Met and p.Gly439Ser) (Figure 1) [4,5]. At last follow-up, fourteen months after the acute episode, the patient was clinically well, with an oral supplementation of 4 meq/kg/day of potassium chloride and 5 mg/kg/day of magnesium element. She was no longer polyuric.

## Discussion

We report here the first case of Gitelman syndrome presenting with the biological features of Fanconi syndrome and an early severe polyuria since the neonatal period. In 1966 Gitelman *et al.* described a familial disorder in three adult female patients who presented with occasional episodes of muscle weakness and tetany [1]. Neither polyuria nor growth retardation were observed. Hypokalemia, hypomagnesemia and hypocalciuria were the biochemical hallmarks of the disease. Since the initial report many cases have been described and the prevalence is estimated to be approximately 1 in 40.000 [5]. Here, we describe a very atypical clinical and biological presentation of Gitelman syndrome in a 6 year old girl.

Most review articles and editorials present Gitelman syndrome as a disease with a relatively mild course, mostly discovered during routine investigations in adolescents or adult patients [6]. Cruz *et al.* reported a cohort of 50 adults with Gitelman syndrome [7]. Most patients being symptomatic, and the most common symptoms were musculoskeletal symptoms (such as cramps, muscle weakness and pain), generalized weakness and salt craving. Polyuria was observed in half of the patients. The authors defined polyuria as voiding at least five times during the daytime. Without quantitative measures, it seems difficult to evaluate the importance of polyuria, and therefore authors may have overestimated its prevalence. Regarding polydipsia, the daily intake in their adult patient cohort was (2.43 ± 1.28 L). Although these values are higher than fluid intake in controls (1.89 ± 0.42 L), it does not seem to be a very pronounced polydipsia for adult patients in whom the acceptable threshold limit for polydipsia is 3 L. Pathophysiologically, polyuria and polydipsia are less pronounced in GS than in classic Bartter syndrome, because only 7% of total salt reabsorption occurs in the distal tubule where the NCCT cotransporter involved in Gitelman syndrome is located. By contrast, in Bartter syndrome, the impaired reabsorption of sodium chloride involves the thick ascending limb of Henle which reabsorbs 30% of filtered NaCl and participates to the generation of the osmotic cortico-papillary gradient [3]. This trend was described in the literature, and in the largest series of Gitelman syndrome [8-10] polyuria was either absent at diagnosis or was not a major symptom. In our case, marked polyuria and polydipsia starting from the neonatal period, with a daily intake of 3 L at the age of 6 years were the main clinical symptoms. As polyuria significantly decreased after the correction of kaliemia in our child, we hypothesized that a concentrating defect concomitant to the severe initial hypokalemia was an aggravating factor to the potentially mild polyuria due to sodium loss observed in Gitelman syndrome. Indeed hypokalemia is known to induce polydipsia and urinary concentration defect by downregulation of aquaporin-2 [11,12].

The initial laboratory findings were more in favor of a proximal tubular defect (sodium and potassium losses, hypophosphatemia, renal tubular acidosis and hypouricemia with high fractional uric acid excretion). Although hypophosphatemia is not common in Gitelman syndrome, Vigano *et al.* showed that even if the bulk of phosphate is reabsorbed in the proximal tubule, there was a tendency towards renal phosphate wasting with mild to moderate hypophosphatemia in their cohort of 12 patients with Gitelman syndrome [13]. In our patient hypophosphatemia was transient suggesting the importance of a long term follow-up of phosphate levels. The plasma acidosis and uric acid loss found initially in our patient could also be part of a tubular proximal defect secondary to the hypokalemia. Indeed, tubular proteinuria has been detected in patients with severe hypokalemia of different etiologies [14] and a transitory proximal dysfunction (tubular proteinuria, phosphaturia uricosuria and aminoaciduria) in patients with hypokalemia associated with renal tubular acidosis [15,16] Nevertheless, as proximal dysfunction in distal renal tubular acidosis is corrected by alkali and potassium supplementation, a participation of acidosis in this dysfunction can not be excluded. Although tubular proteinuria and aminoaciduria were not assessed in our patient, we hypothesize that the transitory proximal dysfunction (acidosis, hypophosphatemia and hypouricemia) is secondary to the severe hypokalemia (1.6 mmol/l).

A few cases of Gitelman syndrome associated with glomerular or tubulointerstitial disease have been reported [17], but to date, no case associated to or with an initial profile of Fanconi syndrome have been described.

## Conclusion

Gitelman syndrome can have varying and sometimes atypical presentations and should be suspected in case of hypokalemic tubular disorders that do not belong to any syndromic entity. This case report emphasizes the need for repeated laboratory tests in undiagnosed tubular disorders, especially not during decompensation episodes. Severe transient polyuria and proximal dysfunction may be observed in case of severe hypokaliemia and this symptom improves after potassium supplementation.

### Consent

Written informed consent was obtained from the patient for publication of this case report and any accompanying images. A copy of the written consent is available for review by the Editor of this journal.

## Abbreviations

NCCT: The thiazide sensitive sodium chloride co-transporter channel.

## Competing interests

None of the authors reports competing interests.

## Authors’ contributions

KB cared for the patient and drafted the manuscript, he read and approved the final manuscript as submitted. OB helped draft the initial manuscript, critically reviewed the manuscript, read and approved the final manuscript as submitted. AG cared for the patient, read and approved the final manuscript as submitted. LH, PN and RS reviewed and revised the manuscript. They read and approved the final manuscript as submitted. LM supervised the *SLC12A3* gene sequencing, read and approved the final manuscript as submitted. RV supervised the SLC12A3 gene sequencing, wrote critical portions of the manuscript related to pathophysiology, read and approved the final manuscript as submitted.

## Pre-publication history

The pre-publication history for this paper can be accessed here:

http://www.biomedcentral.com/1471-2431/14/201/prepub
